# Determinants of long-term outcome in ICU survivors: results from the FROG-ICU study

**DOI:** 10.1186/s13054-017-1922-8

**Published:** 2018-01-18

**Authors:** Etienne Gayat, Alain Cariou, Nicolas Deye, Antoine Vieillard-Baron, Samir Jaber, Charles Damoisel, Qin Lu, Xavier Monnet, Isabelle Rennuit, Elie Azoulay, Marc Léone, Heikel Oueslati, Bertrand Guidet, Diane Friedman, Antoine Tesnière, Romain Sonneville, Philippe Montravers, Sébastien Pili-Floury, Jean-Yves Lefrant, Jacques Duranteau, Pierre-François Laterre, Nicolas Brechot, Karine Chevreul, Morgane Michel, Bernard Cholley, Matthieu Legrand, Jean-Marie Launay, Eric Vicaut, Mervyn Singer, Matthieu Resche-Rigon, Alexandre Mebazaa

**Affiliations:** 1 Department of Anesthesiology, Critical Care and Burn Unit, Hôpitaux Universitaires Saint Louis—Lariboisière, Assistance Publique—Hôpitaux de Paris, Université Paris Diderot—Paris 7, Sorbonne Paris Cité, UMR-S 942, INSERM, Paris, France; 2 Medical Intensive Care Unit, Cochin University Hospital, Assistance Publique—Hôpitaux de Paris, Paris Descartes University, Paris Cardiovascular Research Center-INSERM U970 (PARCC), Paris Sudden Death Expertise Center, Paris, France; 3 Medical Intensive Care Unit, Hôpitaux Universitaires Saint Louis—Lariboisière, Assistance Publique—Hôpitaux de Paris, Université Paris Diderot—Paris 7, Sorbonne Paris Cité, UMR-S 942, INSERM, Paris, France; 4 Intensive Care Unit, University Hospital Ambroise Paré, Assistance Publique—Hopitaux de Paris, 26930 Boulogne-Billancourt, France; 5 Intensive Care Unit, Anaesthesia and Critical Care Department, Saint Eloi Teaching Hospital, Centre Hospitalier Universitaire Montpellier, Montpellier University, Montpellier, France; 6 Multidisciplinary Intensive Care Unit, Department of Anesthesiology and Critical Care Medicine, La Pitié-Salpêtrière Hospital, Assistance Publique Hôpitaux de Paris, UPMC Paris 6, Paris, France; 7 Medical Intensive Care Unit, Bicêtre Hospital, Paris-Sud University Hospitals, Inserm UMR_S999, Paris-Sud University, Le Kremlin-Bicêtre, France; 8 Department of Anesthesiology and Critical Care, Beaujon Hospital, Assistance Publique Hôpitaux de Paris University, Clichy, France; 9 Medical Intensive Care Unit, Hôpital Saint-Louis, ECSTRA Team, Biostatistics and Clinical Epidemiology, UMR 1153 (Center of Epidemiology and Biostatistics Sorbonne Paris Cité, CRESS), INSERM, Université Paris Diderot Sorbonne, Paris, France; 10 Service d’anesthésie et de réanimation, Hôpital Nord, Assistance Publique—Hôpitaux de Marseille, Aix Marseille Université, Marseille, France; 11 Service de Réanimation Médicale, Hôpital Saint-Antoine, Assistance Publique—Hôpitaux de Paris, Université Pierre et Marie Curie, Paris, France; 12 General Intensive Care, Raymond Poincaré University Hosptal, Assistance Publique—Hopitaux de Paris, Garches, France; 13 Department of Anesthesiology and Intensive Care, Cochin University Hospital, Assistance Publique—Hôpitaux de Paris, Paris Descartes University, Paris Cardiovascular Research Center-INSERM U970 (PARCC), Paris Sudden Death Expertise Center, Paris, France; 14 Department of Intensive Care Medicine and Infectious Diseases, Univ Paris Diderot, Sorbonne Paris Cité, Assistance Publique—Hôpitaux de Paris, Hôpital Bichat-Claude, Paris, France; 15 Department of Anesthesiology and Intensive Care, Bichat University Hospital, Assistance Publique—Hôpitaux de Paris, Université Paris Diderot—Paris 7, Sorbonne Paris Cité, Paris, France; 16 Department of Anesthesiology and Intensive Care Medicine, University Hospital of Besancon, 25000 Besancon, France; 17 Department of Anesthesiology, Emergency and Critical Care Medicine, Nimes University Hospital, 30029 Nîmes, France; 18 Physiology Department, EA 2992, Faculté de Médecine de Nîmes, Université Montpellier 1, 30029 Nîmes, France; 19 Département d’Anesthésie-Réanimation, Hôpital de Bicêtre, Université Paris-Sud, Hôpitaux Universitaires Paris-Sud, Assistance Publique—Hôpitaux de Paris, Le Kremlin Bicêtre, Paris, France; 20 Medical–Surgical Intensive Care Unit, Cliniques Saint-Luc, Brussels, Belgium; 21 Medical Intensive Care Unit, Hôpital Pitié-Salpêtrière, Assistance Publique—Hôpitaux de Paris, Sorbonne Pierre-Marie Curie University Paris, INSERM, UMRS_1166-ICAN, Institute of Cardiometabolism and Nutrition and CIC 1421—Paris Est, Paris, France; 22 URC-Eco, Assistance Publique—Hôpitaux de Paris, Sorbonne Paris Cité, Université Paris Diderot, ECEVE, INSERM, Paris, France; 23 Department of Anesthesiology and Critical Care Medicine, Hôpital Européen Georges Pompidou, APHP, Université Paris Descartes, Sorbonne Paris Cite, Paris, France; 24 Service de Biochimie, Hôpitaux Universitaires Saint Louis—Lariboisière, Assistance Publique—Hôpitaux de Paris, Université Paris Diderot—Paris 7, Sorbonne Paris Cité, UMR-S 942, INSERM, Paris, France; 25 Unité de Recherche Clinique, Hôpitaux Universitaires Saint Louis—Lariboisière, Assistance Publique—Hôpitaux de Paris, Université Paris Diderot—Paris 7, Sorbonne Paris Cité, UMR-S 942, INSERM, Paris, France; 26 Bloomsbury Institute of Intensive Care Medicine, University College London, Cruciform Building, Gower St, London, WC1E 6BT UK; 27 Service de Biostatistique et Information Médicale, Hôpitaux Universitaires Saint Louis—Lariboisière, Assistance Publique—Hôpitaux de Paris, Université Paris Diderot—Paris 7, Sorbonne Paris Cité, ECSTRA Team, INSERM, Paris, France; 28 Department of Anesthesiology and Intensive Care, University Paris Diderot, INSERM UMR-S 942, Saint Louis—Lariboisière University Hospitals, 2 rue Ambroise Paré, 75010 Paris, France

**Keywords:** Post-intensive care syndrome, Long-term survival, Biomarkers, Score, Discharge

## Abstract

**Background:**

Intensive care unit (ICU) survivors have reduced long-term survival compared to the general population. Identifying parameters at ICU discharge that are associated with poor long-term outcomes may prove useful in targeting an at-risk population. The main objective of the study was to identify clinical and biological determinants of death in the year following ICU discharge.

**Methods:**

FROG-ICU was a prospective, observational, multicenter cohort study of ICU survivors followed 1 year after discharge, including 21 medical, surgical or mixed ICUs in France and Belgium. All consecutive patients admitted to intensive care with a requirement for invasive mechanical ventilation and/or vasoactive drug support for more than 24 h following ICU admission and discharged from ICU were included. The main outcome measure was all-cause mortality at 1 year after ICU discharge. Clinical and biological parameters on ICU discharge were measured, including the circulating cardiovascular biomarkers N-terminal pro-B type natriuretic peptide, high-sensitive troponin I, bioactive-adrenomedullin and soluble-ST2. Socioeconomic status was assessed using a validated deprivation index (FDep).

**Results:**

Of 1570 patients discharged alive from the ICU, 333 (21%) died over the following year. Multivariable analysis identified age, comorbidity, red blood cell transfusion, ICU length of stay and abnormalities in common clinical factors at the time of ICU discharge (low systolic blood pressure, temperature, total protein, platelet and white cell count) as independent factors associated with 1-year mortality. Elevated biomarkers of cardiac and vascular failure independently associated with 1-year death when they are added to multivariable model, with an almost 3-fold increase in the risk of death when combined (adjusted odds ratio 2.84 (95% confidence interval 1.73–4.65), *p* < 0.001).

**Conclusions:**

The FROG-ICU study identified, at the time of ICU discharge, potentially actionable clinical and biological factors associated with poor long-term outcome after ICU discharge. Those factors may guide discharge planning and directed interventions.

**Trial registration:**

ClinicalTrials.gov NCT01367093. Registered on 6 June 2011.

**Electronic supplementary material:**

The online version of this article (10.1186/s13054-017-1922-8) contains supplementary material, which is available to authorized users.

## Background

Survivors of critical illness will face a period of increased risk of reduced long-term survival and impaired quality of life compared to the general population [1]. This period, lasting several years, is associated with an increased risk of posttraumatic stress, depression, cognitive impairment and physical weakness, all grouped under the entity “post-intensive care syndrome” (PICS) [2].

To reduce the mortality rate of intensive care unit (ICU) survivors, it is important to identify the group of patients who have a higher probability of death in the year following ICU discharge and to recognize the adjustable factors associated with mortality. Although data have been published regarding the long-term outcome of ICU patients, there are no recommendations for the long-term management of these patients. Only experts’ opinions have been published [2, 3]. Some studies have demonstrated that mortality rates among ICU survivors are higher compared to the general population [4–8] and that an ICU stay impacts on patients’ quality of life [9] and disability [10, 11]. Moreover, other studies [5, 6] found that this over-risk of mortality is sustained after 5–15 years of follow-up. Three studies [4, 7, 8] reported a worse survival rate for ICU patients compared to an age-matched control population in the years following ICU discharge. Although we understand that age, comorbidity burden and severity of acute illness are important predictors of late mortality as described previously [12], we know less about clinical and laboratory values at the time of ICU discharge.

The transition of care from ICU to ward and, eventually, to home is a complex process with many challenges. We hypothesized that clinical and biological abnormalities present on the day of ICU discharge are associated with worse long-term outcome. In particular, we hypothesized that ICU survivors are at long risk of increased cardiovascular events, as suggested previously [13]. Among biological abnormalities, we focused on circulating cardiovascular biomarkers, namely N-terminal pro-B type natriuretic peptide (NT-proBNP), high-sensitive troponin I (hs-TnI), bioactive-adrenomedullin (bio-ADM) and soluble-ST2 (sST2). The choice of those four biomarkers was guided by their relative function, with NT-proBNP a marker of cardiac congestion, hs-TnI a marker of cardiac injury, sST2 a marker of cardiac remodeling and bio-ADM a marker of vascular dysfunction.

Accordingly, the FROG-ICU (French and European Outcome reGistry in Intensive Care Units) study aimed to identify clinical and biological (including cardiovascular biomarkers) parameters associated with long-term outcome in ICU survivors.

## Methods

### Study design

FROG-ICU was a prospective, observational, multicenter cohort study in which survivors of critical illness were followed up for up to 1 year post ICU discharge. The study was conducted in France and Belgium in accordance with Good Clinical Practice (Declaration of Helsinki 2002) and Ethical Committee approvals (Comité de Protection des Personnes—Ile de France IV, IRB n°00003835 and Commission d’éthique biomédicale hospitalo-facultaire de l’hôpital de Louvain, IRB n°B403201213352). It is registered on ClinicalTrials.gov (NCT01367093). Patients were included from August 2011 to June 2013. Details of design and methods have been published previously [14]. All patients admitted to any of the participating centers during the recruitment period who met the eligibility criteria and survived their ICU stay had a clinical examination and biological tests performed at discharge from the ICU, and were followed up for 1 year through telephone calls and postal questionnaires at 3, 6 and 12 months.

### Participants

The study involved 21 medical, surgical or mixed ICUs in 14 university hospitals. Inclusion criteria were: invasive mechanical ventilation support for at least 24 h and/or treatment with a vasoactive agent (except dopamine) for more than 24 h. Noninclusion criteria were: age younger than 18 years old; severe head injury (initial Glasgow Coma Scale ≤ 8), brain death or a persistent vegetative state; pregnancy or breastfeeding; transplantation in the past 12 months; moribund patient; and/or no social security coverage. The Ethical Committees waived the need for written consent; all patients and/or next of kin were informed and oral consent was documented in the patients’ medical records by the investigator.

### Study objectives

The primary purpose of the FROG-ICU study was to assess the incidence of all-cause mortality in the year following ICU discharge, and to identify independent factors associated with mortality. The main secondary objective of FROG-ICU was to evaluate the association between circulation cardiovascular biomarkers levels at discharge and 1-year mortality.

### Data collection

Details of data collection have been reported previously [14]. Briefly, clinical and biological data were recorded at admission, during the ICU stay and at discharge from the ICU. In order to explore the mechanisms of clinical abnormalities at ICU discharge associated with subsequent deaths, cardiovascular biomarkers were collected at discharge and measured centrally. These included markers of: cardiac failure (NT-proBNP; Roche Diagnostics GmbH, Mannheim, Germany); cardiac ischemia (hs-TnI; Abbott, Abbott Park, IL, USA); vascular dysfunction [15] (bio-ADM; Adrenomed GmbH, Hennigsdorf, Germany); and cardiac stress (sST2; Eurobio, Critical Diagnostics, San Diego, CA, USA) which prognosticates for cardiovascular death [16, 17]. The deprivation index (FDep) was used as a measure of socioeconomic inequalities in health status. The FDep is based on the patients’ residential zip codes and was specifically developed for the French context using the following four variables to compute a single composite index: median household income, percentage of high school graduates in the population aged ≥ 15 years, percentage of blue-collar workers in the active population and unemployment rate [18].

### Statistical analysis

Results are expressed as median (interquartile range (IQR)) or count (percentage) as appropriate. The primary analysis examining factors associated with 1-year mortality was based on analysis of the clinical and biological variables measured in patients discharged alive from the ICU. Marginal associations between single variables and 1-year mortality were assessed by a Wilcoxon rank-sum test for quantitative variables and the chi-square test for qualitative variables. Multivariable logistic regression was used to determine a set of variables independently associated with 1-year mortality. Variables associated with outcome at a 0.05 level and with less than 20% of missing data were considered within the multivariable model. The log-linearity of the quantitative variables was evaluated systematically, and, if appropriate, variable transformation was performed. Log-linearity of the association between continuous variables and the outcome was checked using a cubic spline and the Wald test. Cutoff values were derived from the plots of the effect according to the value of the variable of interest. Missing values were handled by multiple imputation by chained equations (MICE) [19]. All variables selected for the multivariable model were considered in the imputation model. A total of 51 imputed samples was generated using 15 iterations of the chained equation process. A selection model process was performed using a backward stepwise approach with stopping rules based on a cutoff at 0.05 for *p* values. At each step of the selection, inference was combined from the sets of imputed samples using Rubin’s rules [20]. The existence of any colinearities was observed, and a test of goodness of fit was performed using the Hosmer–Lemeshow test on the complete case model [21]. Measures of association consisted of odds ratios (ORs) and their confidence intervals (CIs) at 95% estimated using Rubin’s rules. The predictive power of the four biomarkers of interest was assessed using receiver operating curve (ROC) analyses. The area under the ROC (AUC) was estimated for each biomarker. For both the clinical model and the clinical model including biomarker information, the AUCs were estimated from the sets of imputed samples using Rubin’s rules. The latter two were compared using the Delong test. As it is now recognized that highlighting a statistically significant association between new biomarkers and patient outcomes is not sufficient to demonstrate the interest of these biomarkers in terms of risk prediction [22–24], we used the proposed methodology of Pencina et al. [23], which has been used in multiple articles of application. The net reclassification improvement (NRI) and integrated discrimination improvement (IDI) of each biomarker added to the full clinical model will be calculated, and comparisons between different biomarkers will be performed [23].

Calculating the number of subjects required was based on the primary endpoint; that is, the risk factors associated with 1-year all-cause mortality. Study of the literature and preliminary studies conducted in December 2009 in 14 participating centers led us to estimate a 1-year mortality after ICU discharge of 18%. To ensure detection with a power of 80% for the detection of binary prognostic factors with a prevalence of 33% and an expected OR of 1.5 in a population with a probability of death in the year following ICU discharge of approximately 18%, 1636 patients were required [25]. Assuming a 10% rate of refusal and/or loss to follow-up, the number of patients to be enrolled was raised to 1800. Finally, since the expected in-ICU mortality rate was 25%, the total number of patients included in the study was 2250. *p* < 0.05 was considered statistically significant. All statistical analyses were performed using R statistical software version 3.1.1 or above (The “R” Foundation for Statistical Computing, Vienna, Austria).

## Results

**Fig. 1:**
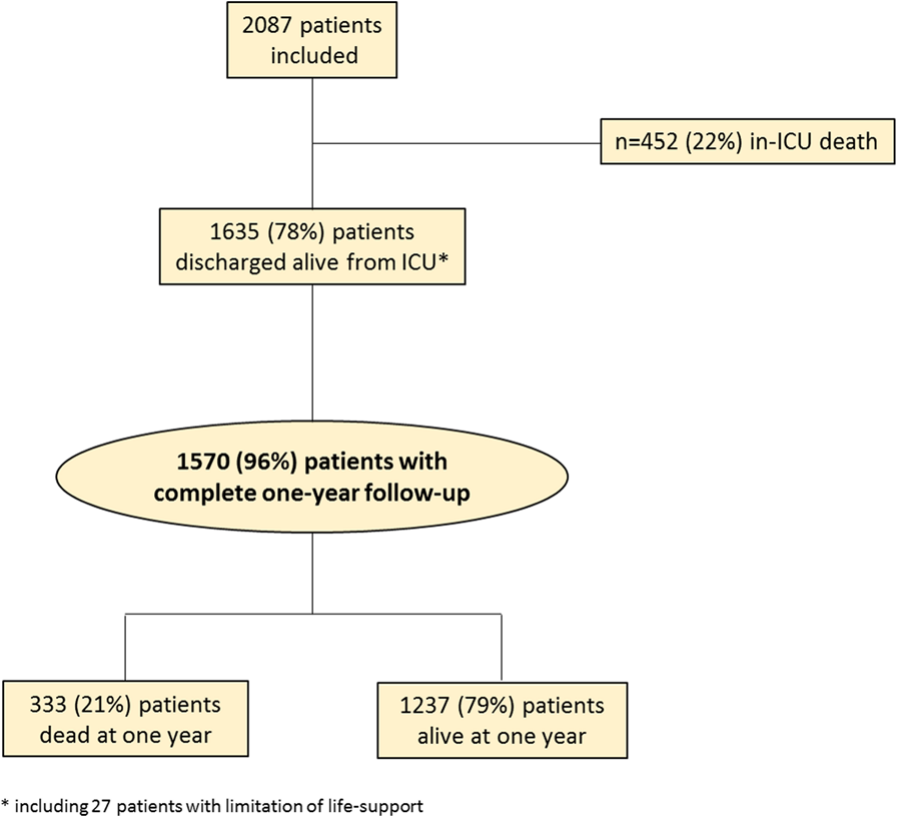
Study flow chart. *Including 27 patients with limitation of life support. ICU intensive care unit

Of the 2087 ICU patients who consented to participate in the FROG-ICU study, 1570 were discharged from the ICU and followed up for 1 year (Fig. 1). Patient characteristics are presented in Table 1. ICU mortality was 22%. Median ICU and hospital lengths of stay for ICU survivors were 12 (IQR 7; 21) and 26 (IQR 15; 43) days, respectively. Details of patients’ comorbidities are presented in Additional file 1: Table S1. The main reasons for ICU admission were septic shock (22%), acute respiratory failure (19%), acute neurological disorder (15%) and out-of-hospital cardiac arrest (8%). On admission, the Sequential Organ Failure Assessment (SOFA) score was 6 (IQR 4; 9) and the Simplified Acute Physiologic Score (SAPS) II was 46 (IQR 34; 59).

**Table 1:** Patient characteristics

Variable	% of missing value	Studied patients	1 year post ICU		*p* value
		(*n* = 1570)	Survivors (*n* = 1237)	Nonsurvivors (*n* = 333)	
Age (years)	0.0	61 (49; 73)	58 (47; 70)	71 (61; 79)	< 0.01
Male gender	0.0	1000 (63.7)	782 (63%)	218 (66%)	0.48
BMI (kg/m^2^)	37.5	26 (23; 31)	26 (23; 31)	26 (23; 31)	0.96
Charlson score	0.1	3 (1; 4)	2 (1; 4)	4 (3; 6)	< 0.01
Deprivation index (FDep)	10.8	−0.6 (−1.6; 0.3)	−0.6 (−1.6; 0.3)	−0.6 (−1.5; 0.4)	0.44
SOFA score at admission	37.2	6 (4; 9)	7 (4; 10)	8 (5; 11)	0.11
SAPS II score at admission	0.1	46 (34; 59)	45 (33; 58)	51 (40; 65)	< 0.01
Main cause of admission					< 0.01
Septic shock	0.1	339 (22%)	244 (20%)	95 (29%)	
Acute respiratory failure		303 (19%)	230 (19%)	73 (22%)	
Acute neurological disorder		241 (15%)	210 (17%)	31 (9%)	
Out-of-hospital cardiac arrest		117 (8%)	102 (8%)	15 (5%)	
In-ICU management					
In-ICU LOS (days)	0.0	12 (7; 21)	12 (7; 20)	13 (7; 24)	0.03
In-hospital LOS (days)	0.1	26 (15; 43)	25 (15; 43)	28 (16; 47)	0.05
Tracheotomy	0.0	241 (15%)	181 (15%)	60 (18%)	0.13
RRT	0.0	286 (18%)	202 (16%)	84 (25%)	< 0.01
Inotrope/vasopressor	0.0	1151 (73%)	888 (72%)	263 (79%)	< 0.01
RBC	0.0	676 (43%)	490 (40%)	186 (56%)	< 0.01
FFP	0.0	236 (15%)	171 (14%)	65 (20%)	< 0.01
Status at discharge					
SBP (mmHg)	12.9	125 (111; 139)	125 (112; 139)	122 (108; 139)	0.03
DBP (mmHg)	16.7	68 (59; 76)	69 (60; 77)	64 (55; 73)	< 0.01
HR (bpm)	14.1	90 (79; 101)	90 (79; 100)	89 (79; 101)	0.41
Atrial fibrillation	10.0	297 (21%)	265 (21%)	32 (21.2)	0.96
Temperature (°C)	10.3	37.1 (36.8; 37.5)	37.1 (36.8; 37.5)	37 (36.6; 37.4)	< 0.01
Sodium (mmol/l)	3.1	139 (136; 142)	139 (136; 142)	139 (136; 142)	0.6
Potassium (mmol/l)	8.3	3.9 (3.6; 4.2)	3.9 (3.6; 4.2)	4.0 (3.6; 4.2)	0.36
Creatinine (μmol/l)	3.7	66 (51; 95)	64 (50; 87)	80 (57; 131)	< 0.01
eGFR (ml/min/1.73 m^2^)	3.7	91 (51.2; 110)	110 (75; 146)	79 (46; 119)	< 0.01
Lactate (mmol/l)	58.3	1.0 (0.7; 1.3)	1.0 (0.7; 1.3)	1.1 (0.8; 1.4)	< 0.01
WBC count (/mm^3^)	13.6	9600 (7015; 13,100)	9500 (7000; 12,952)	10050 (7342; 13,962)	0.04
Hemoglobin (g/dl)	13.6	10.0 (9.0; 11.2)	10.2 (9.1; 11.3)	9.6 (8.7; 10.6)	< 0.01
Platelets count (/mm^3^)	12.9	291,500 (181,750; 432,500)	308,500 (191,000; 457,000)	240500 (137,500; 347,750)	< 0.01
Bilirubin (mmol/l)	63.2	11 (7; 20)	10 (7; 19)	14 (9; 36)	< 0.01
Glycemia (mmol/l)	16.8	6.8 (5.7; 8.3)	6.7 (5.7; 8.2)	7.1 (5.9; 8.7)	0.01
Total protein (g/L)	18.6	62 (56; 69)	63 (57; 69)	60 (52; 66)	< 0.01

Results expressed as count (percentage) or median (interquartile range)

BMI body mass index, *SOFA* Sequential Organ Failure Assessment, *SAPS* Simplified Acute Physiology Score, *ICU* intensive care unit, *LOS* length of stay, *RRT* renal replacement therapy, *RBC* red blood cell transfusion, *FFP* fresh frozen plasma transfusion, *SBP* systolic blood pressure, *DBP* diastolic blood pressure, *HR* heart rate, *eGFR* estimated glomerular filtration rate, *WBC* white blood cell

Clinical and biological characteristics at the time of ICU discharge were generally in the normal range (Table 1), except for hemoglobin (median value 10 g/dl). Patients were mostly discharged to a ward (*n* = 976, 50%) or step-down unit (*n* = 269, 14%).

### Determinants of 1-year survival after ICU discharge

Of the 1570 ICU survivors, 333 (21%) died during the year following ICU discharge, including 123 (8%) during the index hospitalization (Additional file 1: Figure S1). Univariate analysis revealed that the 333 nonsurvivors at 1 year post ICU discharge had a greater degree of illness severity at ICU admission and more comorbidities (Table 1, Additional file 1: Table S1). One-year nonsurvivors were more likely to have septic shock as the cause of admission. While in the ICU, 1-year nonsurvivors required more renal replacement therapy, inotropes/vasopressors and transfusion than survivors. On ICU discharge, nonsurvivors had lower blood pressure and residual organ dysfunction than survivors. Yet renal function was more profoundly altered in nonsurvivors with a higher serum creatinine and lower eGFR at ICU discharge (Table 1).

**Fig. 2:**
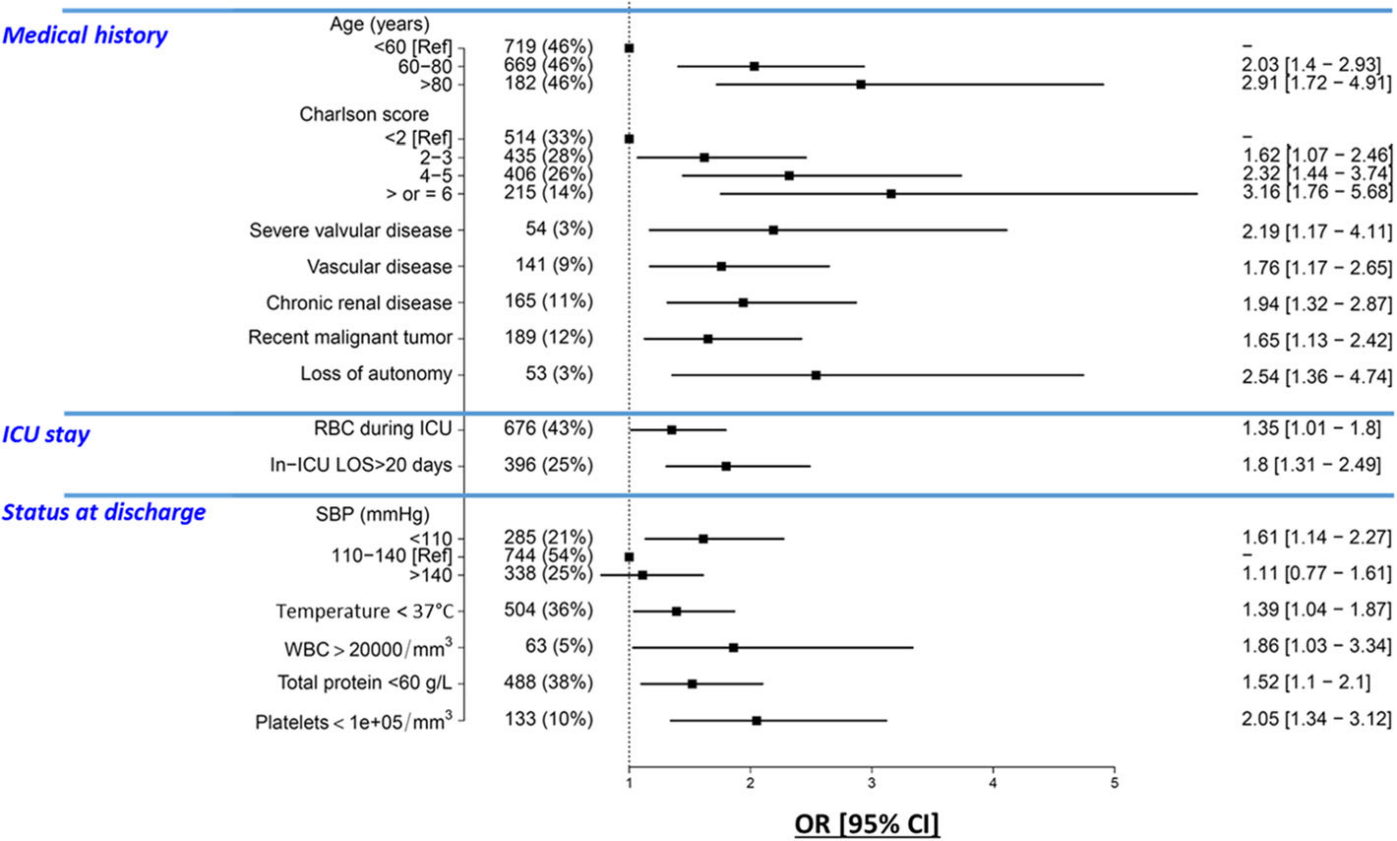
Clinical predictors of 1-year post-ICU survival. Area under the ROC curve of the multivariable model including the 14 variables is 0.787 (95% CI 0.759–0.815). RBC red blood cell transfusion, ICU intensive care unit, LOS length of stay, SBP systolic blood pressure, WBC white blood cell, Ref reference, OR odds ratio, CI confidence interval

Multivariable analysis identified 14 independent predictors of post-ICU survival (Fig. 2). Odds ratios of significantly associated variables are presented in Additional file 1: Table S2. Linearity of the association between continuous variables in the multivariable model and the outcome is depicted in Additional file 1: Figure S2. The area of the ROC curve for the multivariable model was 0.787 (95% CI 0.759–0.815). Age and comorbidities (Charlson comorbidity score, vascular disease, severe valvular disease, chronic kidney diseases, cancer and loss of autonomy) were associated with a greater 1-year risk of death. At ICU discharge, five clinical variables (low values of systolic blood pressure, body temperature, total protein and platelet counts, and a high white blood cell count) were associated with an increased post-ICU risk of death. With respect to their ICU stay, red blood cell transfusion and prolonged ICU length of stay were associated with higher risk of 1-year post-ICU mortality. Of note, AUCs of SOFA at admission and SAPS II were 0.574 (95% CI 0.531–0.619) and 0.605 (95% CI 0.572–0.64) respectively; both were significantly lower than the AUC of the clinical score.

### Association between cardiovascular biomarkers at discharge and 1-year survival after ICU discharge

**Fig. 3:**
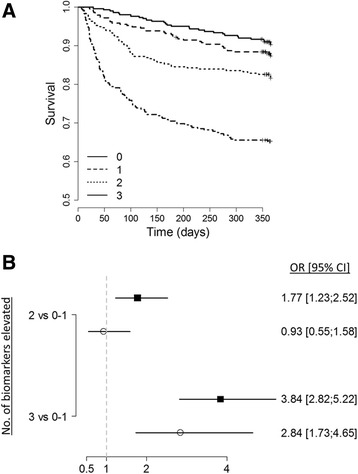
Performance of cardiovascular biomarkers at ICU discharge to predict 1-year post-ICU survival. **a** Kaplan–Meier curves of patients discharged alive from the ICU according to the number of cardiovascular biomarkers elevated at discharge from the ICU. **b** Odds ratios for the risk of 1-year mortality according to the number of cardiovascular biomarkers elevated at discharge from the ICU. Biomarkers included NT-proBNP, sST2 and bio-ADM. Nonadjusted OR are presented as black squares and OR adjusted for the 14 variables of the multivariable model as white circles. OR odds ratio, CI confidence interval

At the time of ICU discharge, 1-year nonsurvivors had elevated levels of all measured cardiovascular biomarkers (Table 2). As depicted in Additional file 1: Figure S3, the association between the level of biomarkers at discharge and the outcome was not linear in all cases. After dichotomization according to the median value, elevated biomarkers of cardiac (NT-proBNP, sST2) and vascular (bio-ADM) failure were independently associated with 1-year death when they are added to the multivariable model, with an almost 3-fold increase in the risk of death when combined (adjusted OR 2.84 (95% CI 1.73–4.65), *p* < 0.001) (Fig. 3). Of note, the association between elevated hs-TnI and 1-year mortality did not remain significant after adjustment. Although only NT-proBNP, bio-ADM and sST2 significantly improve the *c*-statistic of the clinical model, reclassification analyses showed that all cardiovascular biomarkers, including hs-TnI, improve predictive power of the multivariable model (Table 2).

**Table 2:** Performance of cardiovascular biomarkers measured at ICU discharge for the prediction of 1-year post-ICU survival

	NT-proBNP (pg/ml)	hs-TnI (pg/ml)	bio-ADM (pg/ml)	sST2 (ng/ml)
Normal value	< 300	< 14	< 43	< 23.6 for men/< 16.0 for women
Missing data (%)				
Median (IQR) at discharge				
All patients (*n* = 1570)	541 (149; 2073)	11.2 (4.2; 39.2)	33.3 (20.2; 60.5)	122.2 (73.1; 208.8)
1-year survivor (*n* = 1237)	464 (127; 1681)	9.7 (3.7; 33.8)	30.5 (18.7; 52.3)	112.5 (67.9; 188.6)
1-year nonsurvivor (*n* = 333)	1471 (371; 5719)	18.5 (7.6; 74.7)	50.4 (28.5; 107.9)	189.1 (102.4; 301.2)
Association with prognosis (OR (95% CI) of biomarker > median)				
Univariate analysis	2.50 (1.85–3.38)	2.03 (1.51–2.73)	2.52 (1.86–3.42)	2.44 (1.81–3.30)
Multivariable analysis	2.05 (1.33–3.18)	1.41 (0.94–2.13)	1.61 (1.06–2.45)	1.53 (1.01–2.33)
AUC of ROC curve (95% CI)				
Biomarker alone	0.659 (0.619–0.699)*	0.625 (0.588–0.663)*	0.672 (0.635–0.711)*	0.657 (0.618–0.697)*
Biomarker + clinical score	0.794 (0.766–0.823)**	0.789 (0.759–0.817)	0.794 (0.766–0.822)	0.800 (0.773–0.827)**
NRI of biomarkers added to the full clinical model				
% Events to higher risk	44.3	37.4	43.4	42.5
% Nonevents to higher risk	14.8	16.1	15.7	17.2
% Events to lower risk	55.7	62.6	56.6	57.5
% Nonevents to lower risk	85.2	83.9	84.3	82.8
Total NRI for events (95% CI)	−0.115 (−0.263 to 0.033)	−0.253 (−0.397 to −0.109)	−0.133 (−0.281 to 0.015)	−0.149 (−0.296 to −0.003)
Total NRI for nonevents (95% CI)	0.705 (0.650–0.759)	0.678 (0.622–0.734)	0.686 (0.631–0.742)	0.656 (0.598–0.714)
Total cNRI (95% CI)	0.590 (0.433–0.747)	0.425 (0.271–0.580)	0.554 (0.396–0.711)	0.507 (0.349–0.664)
IDI of biomarkers added to the full clinical model				
Events to higher risk	0.014	–0.009	0.018	0.005
Nonevents to lower risk	0.023	0.016	0.024	0.02
Total (95% CI)	0.036 (0.019–0.054)	0.007 (0–0.014)	0.041 (0.023–0.06)	0.024 (0.009–0.04)

*NT-proBNP* N-terminal pro-B type natriuretic peptide, *hs-TnI* hyper-sensitive troponin I, *bio-ADM* bio-adrenomedullin, *sST2* soluble ST2, *IQR* interquartile range, *AUC* area under the curve, *OR* odds ratio, *ROC* receiver operating curve, *CI* confidence interval, *NRI* net reclassification improvement, *IDI* integrative discrimination improvement

*p < 0.05 corresponding to Wilcoxon test comparing survivors to nonsurvivors

**p < 0.05 corresponding to DeLong test comparing *c*-statistics of multivariate clinical model without (see Fig. 2) and with inclusion of the biomarker

## Discussion

The FROG-ICU study confirmed the substantial number of vulnerable patients among ICU survivors. More importantly, FROG-ICU identified clinical and biological factors at the time of ICU discharge that were associated with an increased risk of long-term death.

We found that the 1-year mortality rate in ICU survivors was roughly 20%, a figure comparable to that already described [8, 26–29]. The FROG-ICU study confirmed that increasing age and number of comorbidities are independently associated with an increased long-term risk of death [30]. In contrast to previous findings [31], with the exception of blood transfusion and prolonged length of ICU stay, we found no “in-ICU” factor was associated with an increased risk of post-ICU death. Indeed, the reason for ICU admission, illness severity scores at admission and/or use of invasive therapy, factors known to be associated with ICU mortality, were not associated with worse long-term outcomes in our 1570 consecutive ICU survivors, as described recently [32].

A major strength of the FROG-ICU study is the provision of a comprehensive clinical and biological evaluation of patients at the time of ICU discharge to assess risk prediction for subsequent poor outcomes. FROG-ICU demonstrated that hypotension and symptoms of persisting inflammation (abnormal temperature, protein, platelet and WBC count) were risk factors for a poor post-ICU outcome. FROG-ICU further showed that elevated biomarkers of impaired cardiac (NT-proBNP and sST2) and vascular (bio-ADM) function strikingly improved the prediction of post-ICU risk of death. Altogether, these data demonstrate that evidence of cardiovascular and/or inflammation abnormalities on ICU discharge is associated with, and likely leads to, a poor post-ICU outcome. Specific causes of death need to be ascertained but may be related to accelerated atheroma and plaque formation in the heart, brain or other organs, or repeated bouts of infection related to immunosuppression resulting from persisting inflammation. Those results are consistent with other work suggesting that the level of residual inflammation at discharge for patients with sepsis is associated with subsequent mortality [33].

### Limitations of the study

Sixty-five (4%) patients discharged alive from the ICU were not assessed at 1 year. Although the number is small, this could have affected the accuracy of our results. We cannot assess the risk of readmission after ICU discharge as this information was not recorded prospectively. More broadly, we had no information on patient management (e.g., drug therapy, rehabilitation, psychologist support) after ICU discharge. This may also have contributed to patient vulnerability and needs to be further explored. In addition, while we described clinical and biological variables independently associated with 1-year mortality in ICU survivors, other important parameters need to be considered when discharging a patient from the ICU, such as the amount of nursing care. Some potential predictors of post-ICU outcome were not considered in the present study; in particular, only comorbidities were considered but no frailty score. Moreover, because of the French law, we were not allowed to include patients with no social security coverage, which may limit the external validity of our results. Biological collection was performed when the patient physically left the ICU and not at the time the patient was considered dischargeable from the ICU, which is more tightly linked to the physiologic status of the patient. However, our approach reflects the real-life management of ICU discharge. Although the study was multicentric and conducted in two European countries, only one center outside France included patients; this may limit the external validity of our results. Finally, despite the fact that a sample size calculation was performed, factors that were weakly associated with the 1-year risk of death could not be identified due to insufficient study power. Of note, the main aim of the study was to identify an explanatory model. Thus, the objective of our variable selection procedure was to identify the factors most strongly associated with mortality at 1 year and not to establish a prognostic score that would have to be validated.

## Conclusions

Our findings suggest recommending a comprehensive clinical examination and targeted biological testing, including biomarker measures in ICU survivors, to guide personalized discharge long-term planning. Future trials should assess whether actions targeting the pathophysiology underlying the abnormal cardiac or vascular biomarkers may translate into improved post-ICU outcomes. In summary, the FROG-ICU study confirmed the striking prevalence of death at 1 year after ICU discharge. The FROG-ICU study further identified clinical and biological factors that may guide personalized discharge planning.

## Additional file

Additional file 1: Figure S1. showing Kaplan–Meier curves for 1-year mortality after discharge from the ICU, **Figure S2.** showing plots of restricted cubic spline of continuous variables included in the multivariable model, **Figure S3.** showing plots of restricted cubic spline of continuous variables included in the multivariable model, **Table S1.** presenting details on comorbidities and chronic treatment, and **Table S2.** presenting ORs (with 95% CI) for variables significantly associated with 1-year mortality in univariate analysis and in multivariable analysis (DOCX 110 kb)

## Abbreviations

AUC: Area under the curve
bio-ADM: Bio-adrenomedullin
CI: Confidence interval
eGFR: Estimated glomerular filtration rate
FROG-ICU: French and European Outcome reGistry in Intensive Care Units
hs-TnI: Hyper-sensitive troponin I
ICU: Intensive care unit
IDI: Integrative discrimination index
IQR: Interquartile range
NRI: Net reclassification index
NT-proBNP: N-terminal pro-B type natriuretic peptide
OR: Odds ratio
PICS: Post-intensive care syndrome
ROC: Receiver operating curve
SAPS: Simplified Acute Physiologic Score
SOFA: Sequential Organ Failure Assessment
sST2: Soluble ST2
WBC: white blood cell
