# Supplementary material for: Determinants of long-term outcome in ICU survivors: results from the FROG-ICU study
Source: Crit Care. 2018 Jan 18;22:8. doi: 10.1186/s13054-017-1922-8 (PMC5774139; doi:10.1186/s13054-017-1922-8)
Supplement: Additional file 1: Figure S1. — showing Kaplan–Meier curves for 1-year mortality after discharge from the ICU, Figure S2. showing plots of restricted cubic spline of continuous variables included in the multivariable model, Figure S3. showing plots of restricted cubic spline of continuous variables included in the multivariable model, Table S1. presenting details on comorbidities and chronic treatment, and Table S2. presenting ORs (with 95% CI) for variables significantly associated with 1-year mortality in univariate analysis and in multivariable analysis (DOCX 110 kb) [file 13054_2017_1922_MOESM1_ESM.docx]

**
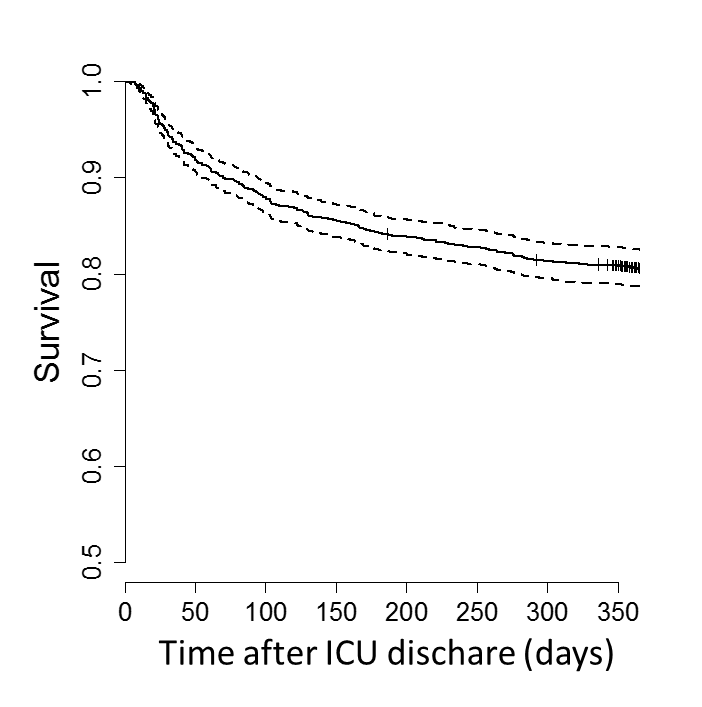
**

**Figure S1.** Kaplan-Meier curves for one-year mortality after discharge from ICU

The straight line indicates instantaneous risk of death while the dashed lines correspond to confidence interval of this risk.

**Figure S2**. Plots of restricted cubic spline of continuous variables included in the multivariable model.

**Figure S3**. Plots of restricted cubic spline of cardiovascular biomarkers considered in the study

Dashed vertical lines represent the median value for each biomarkers.

Abbreviation: NT-proBNP, N-Terminal pro B-type natriuretic peptide. hs-TnI, hyper-sensitive troponin I. bio-ADM, bio-adrenomedullin. sST2, soluble ST2.

| **Table S1**. Details on comorbidities and chronic treatment | | | |  |
| --- | --- | --- | --- | --- |
| **Variables** | **All patients** | **One-year post-ICU** | | **p value** |
|  | **(n=1570)** | **Survivors (n=1237)** | **Non-survivors (n=333)** |  |
| **Comorbidities** |  |  |  |  |
| Chronic heart failure | 108 (7%) | 65 (5%) | 43 (13%) | <0.0001 |
| History of hypertension | 644 (41%) | 457 (37%) | 187 (57%) | <0.0001 |
| Diabetes mellitus | 274 (18%) | 185 (15%) | 89 (27%) | <0.0001 |
| Dyslipidemia | 302 (19%) | 214 (17%) | 88 (27%) | 0.0002 |
| Obesity | 167 (11%) | 124 (10%) | 43 (13%) | 0.13 |
| Coronary artery disease | 165 (11%) | 129 (10%) | 61 (18%) | 0.0001 |
| Prior stroke | 64 (4%) | 41 (3%) | 23 (7%) | 0.005 |
| Peripheral vascular disease | 141 (9%) | 85 (7%) | 56 (17%) | <0.0001 |
| Severe valvular disease | 54 (3%) | 29 (2%) | 25 (8%) | <0.0001 |
| Pulmonary embolism | 43 (3%) | 29 (2%) | 14 (4%) | 0.085 |
| Atrial fibrillation/flutter | 160 (10%) | 104 (8%) | 56 (17%) | <0.0001 |
| Pulmonary hypertension | 17 (1%) | 11 (1%) | 6 (2%) | 0.23 |
| Baseline LVEF (%) | 45 [35;60] | 50 [35.5;60] | 40 [32.5;50] | 0.15 |
| COPD | 173 (11%) | 123 (10%) | 50 (15%) | 0.010 |
| Chronic kidney disease | 165 (11%) | 91 (7%) | 74 (22%) | <0.0001 |
| Chronic liver disease | 100 (6%) | 68 (6%) | 32 (10%) | 0.008 |
| Active recent malignant tumor | 189 (12%) | 118 (10%) | 71 (21%) | <0.0001 |
| Depression | 205 (13%) | 171 (14%) | 34 (10%) | 0.098 |
| Anemia | 23 (2%) | 18 (1%) | 5 (2%) | 1 |
| HIV/AIDS | 39 (3%) | 32 (3%) | 7 (2%) | 0.84 |
| Dysthyrodism | 101 (6%) | 72 (6%) | 29 (9%) | 0.059 |
| Chronic inflammatory disease | 61 (4%) | 41 (3%) | 20 (6%) | 0.036 |
| Cognitive dysfunction | 25 (2%) | 16 (1%) | 9 (3%) | 0.082 |
| Loss of autonomy* | 53 (3%) | 29 (2%) | 24 (7%) | <0.0001 |
| Alcohol abuse | 269 (17%) | 212 (17%) | 57 (17%) | 1 |
| Smoking | 446 (29%) | 363 (29%) | 83 (25%) | 0.12 |
| Other psychiatric disorder | 77 (5%) | 66 (5%) | 11 (3%) | 0.13 |
| Abbreviations: COPD, chronic obstructive pulmonary disease. LVEF, left ventricular ejection fraction. HIV, human immunodeficiency virus. AIDS, acquired immuno-deficiency syndrome | | | | |

* Loss of autonomy was defined by the need for the patient to have assistance in all the acts of his daily life (clothing, food, toilet).

| **Table S2**. Odds ratio for variables significantly associated with one-year mortality in univariate analysis and in multivariable analysis (with 95% confidence interval) | | |
| --- | --- | --- |
|  | **Univariate analysis** | **Multivariable analysis** |
|  | **OR [95% CI]** | **OR [95% CI]** |
| ***Medical history*** |  |  |
| Age (years) |  |  |
| <60 [Ref] | - | - |
| 60-80 | 3.60 [2.69 - 4.8] | 2.03 [1.40 - 2.93] |
| >80 | 6.54 [4.37 - 9.77] | 2.91 [1.72 - 4.91] |
| Charlson score |  |  |
| <2 [Ref] | - | - |
| 2-3 | 3.28 [2.37 - 4.56] | 1.62 [1.07 - 2.46] |
| 4-5 | 5.85 [4.06 - 8.43] | 2.32 [1.44 - 3.74] |
| > or = 6 | 10.86 [6.94 - 16.98] | 3.16 [1.76 - 5.68] |
| Severe Valvular Failure | 3.40 [1.96 - 5.89] | 2.19 [1.17 - 4.11] |
| Chronic heart failure | 2.69 [1.79 - 4.04] |  |
| Vascular disease | 2.76 [1.92 - 3.96] | 1.76 [1.17 - 2.65] |
| Diabetes mellitus | 2.09 [1.57 - 2.79] |  |
| History of hypertension | 2.21 [1.73 - 2.83] |  |
| Prior stroke | 2.18 [1.29 - 3.68] |  |
| Dyslipidemia | 1.73 [1.30 - 2.30] |  |
| Coronary artery disease | 1.73 [1.16 - 2.59] |  |
| Atril fibrillation/flutter | 2.22 [1.56 - 3.15] |  |
| COPD | 1.61 [1.13 - 2.29] |  |
| Chronic liver disease | 1.84 [1.19 - 2.85] |  |
| Chronic kidney disease | 3.62 [2.59 - 5.07] | 1.94 [1.32 - 2.87] |
| Recent malignant tumor | 2.59 [1.87 - 3.58] | 1.65 [1.13 - 2.42] |
| Loss of autonomy | 3.25 [1.87 - 5.67] | 2.54 [1.36 - 4.74] |
|  |  |  |
| ***ICU stay*** |  |  |
| SAPS 2 | 1.02 [1.01 - 1.03] |  |
| Septic shock | 1.63 [1.24 - 2.15] |  |
| RBC | 1.93 [1.51 - 2.46] | 1.35 [1.01 - 1.80] |
| FFP | 1.51 [1.10 - 2.07] |  |
| Inotrope/vasopressor | 1.48 [1.10 - 1.98] |  |
| RRT | 1.73 [1.29 - 2.31] |  |
| In-ICU LOS > 20 days | 1.47 [1.13 - 1.92] | 1.80 [1.31 - 2.49] |
|  |  |  |
| ***Status at discharge*** |  |  |
| Atrial fibrillation | 1.01 [0.67 - 1.53] |  |
| Hemoglobine (g/L) | 0.82 [0.75 - 0.89] |  |
| Systolic blood pressure (mmHg) | |  |
| <110 | 1.78 [1.30 - 2.44] | 1.61 [1.14 - 2.27] |
| 110 - 140 [Ref] | - | - |
| >140 | 1.31 [0.95 - 1.81] | 1.11 [0.77 - 1.61] |
| DBP (for 10 mmHg) | 0.78 [0.70 - 0.86] |  |
| Temperature <37°C | 1.71 [1.32 - 2.22] | 1.39 [1.04 - 1.87] |
| eGFR (for 10 ml/min) | 0.95 [0.92 - 0.97] |  |
| WBC > 20000/mm3 | 2.40 [1.42 - 4.06] | 1.86 [1.03 - 3.34] |
| Total protein <60 g/L | 1.85 [1.41 - 2.43] | 1.52 [1.10 - 2.10] |
| Platelets < 100000/mm3 | 2.66 [1.82 - 3.87] | 2.05 [1.34 - 3.12] |
| Glycemia (for 1 mmol/L) | 1.06 [1.01 - 1.11] |  |
| Abbreviations: SOFA, sequential organ failure assessment. SAPS, simplified acute physiology score. ICU, intensive care unit. LOS, length of stay. RRT, renal replacement therapy. RBC, red blood cell transfusion. FFP, fresh frozen plasma transfusion. SBP, systolic blood pressure. DBP, diastolic blood pressure. HR, heart rate. eGFR, estimated glomerular filtration rate. WBC, white blood cells | | |
